# Impact of Pain Intensity on Relationship Quality Between Couples Where One Has Back Pain

**DOI:** 10.1111/pme.12366

**Published:** 2014-01-21

**Authors:** Arani Vivekanantham, Paul Campbell, Christian D. Mallen, Kate M. Dunn

**Affiliations:** ^1^ Arthritis Research UK Primary Care Centre, Primary Care SciencesKeele UniversityKeeleStaffordshireUK

**Keywords:** Pain, Relationship Quality, Depression, Spouse, Partner

## Abstract

**Objectives:**

To investigate associations of pain intensity in those with long‐term back pain, with their partners' rating of key constructs of relationship quality: cohesion (activities together), consensus (affection, sexual relations), satisfaction (conflict, regrets).

**Methods:**

Self‐report questionnaires on relationship quality (partner‐rated), depression (partner‐rated), relationship length, and pain intensity (patient‐rated) were collected from back pain patients and their partners (N = 71). Linear regression was carried out to test for associations, standardized coefficients (β) and 95% confidence intervals (95% CI) are reported.

**Results:**

There was no main effect between patient pain intensity and partner rating of relationship quality. However, partner ratings of relationship quality were lower if the partner reported increasing depressive symptoms. Adjusting for the effects of partner depression show that ratings of consensus (affection, sexual relations) from partners were actually higher with increasing levels of pain intensity in patients (β 0.54, 95% CI 0.17 to 0.90, *P* < 0.01). Furthermore lower ratings of consensus were reported where patient pain intensity interacted with partner depression (β −0.11, 95% CI—0.19 to −0.03, *P* < 0.05).

**Conclusions:**

These findings illustrate the association of pain outcomes beyond the patient within a primary care sample. Moderators of the responses about the relationship construct of consensus generated by partners appear to be partners' own level of depressive symptoms and whether their depressive symptoms are associated with the patients' pain intensity. Consultations should consider the social context of patients with pain.

## Introduction

Long‐term back pain is a common and major health concern, with a lifetime prevalence estimate of over 70% in industrialized nations [1]. The disability associated with this pain can have widespread effects on both the economy, due to the extensive health care costs and the absences from work [2,3], as well as on the individual and their family [4,5].

It is generally accepted that the experience of back pain is shaped at a biopsychosocial level [6,7]. One area of research interest is the social impact of back pain, in particular the influence of patients' pain and related outcomes on their partners' (e.g., spouse) distress and relationship quality [8,9]. Research shows chronic pain can have a negative impact on the relationship quality between patient and partner, and this can have a reciprocal influence on patient outcome [10,11]. Historically, theoretical explanation of these effects rested on operant behavioral principles whereby patient pain behavior would elicit a response from the partner (e.g., sympathetic solicitousness response or a negative punishing response) which in turn would reinforce patient pain behavior [12].

However, more recent research has revealed a more nuanced understanding of a complex relationship between partners where one has pain. For example Cano et al. [13,14] showed incongruence between patient and partner ratings of pain and disability. Explanations for this incongruence were partly explained by the level of satisfaction of the relationship between partners, suggesting that relationship quality is an important factor. Furthermore, despite the tendency of previous research to generally show a detrimental effect of patient pain and disability on the relationship quality between couples [5,8,11], a recent study found patients with long‐term back pain increased the ratings of their relationship quality as their pain intensity increased after adjustment for depression [15]. Recently Cano and Leong [16] proposed a theoretical model that can account for such accord and discord between pain patients and their partners. They propose that patient pain behaviors are internally processed (decoded) by the partner and evaluated based on empathy, motivation, and emotion. This in turn leads to a behavioral response of the partner, which may be empathetic or unempathetic, which in turn, cyclically influences future pain behavior. This is suggestive that relationship quality and the emotional state (e.g., depression) between partners are key factors in explaining the impact of pain on the partner. Certainly research in the broader field of relationships (e.g., marital discord model) has shown that determinants of discord and depression within a relationship can be influenced by many factors [17,18]. Aspects such as spousal criticism and blame, disruption to scripted routines and other idiosyncratic marital stressors can lead to depression, many of which could be influenced by the presence of pain and disability in a partner. However, these aspects can be offset by the emotional impact of such disruption, as well as the level of cohesion and intimacy a couple will share, again stressing the importance of understanding the interplay of emotional state and relationship quality. Indeed in a recent review of the pain and couple literature, Leonard et al. [11] discusses the need to gather greater detailed knowledge about what specific constructs of relationship quality are influenced by pain in order to better develop treatments that involve partners/spouses.

In this current study, we consider the association between patients' reported pain intensity and their partners' rating of relationship quality (cohesion, consensus, satisfaction) to ascertain whether patient pain intensity associates with judgements of the relationship quality in partners. Consistent with previous literature [11], we hypothesize that increases in patient‐reported pain intensity will associate with lower levels of reported relationship quality in their partners. In addition, in line with the theoretical model of Cano and Leong [16], and the marital discord model [17] outlined above, we further hypothesize that the relationship between pain intensity and relationship quality will be contingent on the mood state of the partner, and whether partner mood is associated also with pain intensity. Therefore, we aim to test direct associations between patient pain intensity and the partners' reported relationship quality and also consider the moderation effect that partner's mood may have on these associations.

## Method

### Design and Setting

This is a cross‐sectional study nested within a larger longitudinal follow‐up study of patients seeking primary health care for low back pain (LBP).

### Participants and Procedures

**Figure 1:**
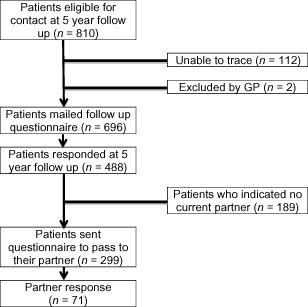
Flow diagram of patient and partner recruitment.

Full details of the original study can be found elsewhere [19,20]. In brief, at the baseline stage, patients who consulted their general practitioner (GP) for LBP were invited to take part. Recruitment was carried out in eight general practices in North Staffordshire and Central Cheshire in England. The patients were identified through the use of Read Codes indicating a primary care consultation for LBP. Read codes are a common method for the computerized recording of morbidity in UK primary care and are most often entered by the patients' GP at the time of consultation [21,22]. The codes selected were intended to include all cases of nonspecific LBP. Patients with codes indicating a red flag diagnosis (e.g., cauda equina syndrome, significant trauma, ankylosing spondylitis, cancers) were excluded [19,20]. In total, 810 patients formed the potential cohort for follow up at 5 years (the time of this study's data collection). However, at 5‐year follow up, 112 patients could not be traced as they had moved practices, and two patients were judged by their GPs as unsuitable to take part due to other illnesses (e.g., dementia). Therefore the eligible cohort for the follow‐up stage was 696 patients. This current study's sample was identified within this wider database. Patients who responded and indicated they had a current partner (a partner was defined as a husband or wife or the person whom they live with) were included. All patients who responded and indicated they had a partner (N = 299) were sent a questionnaire for themselves, and a separate questionnaire to pass on to their partners (see Figure 1 for a flow diagram of the recruitment procedure). It was at the discretion of the patient to pass on the partner questionnaire to their partner. Partners could then return the questionnaire directly to the research team using a stamped addressed envelope.

Ethical approval was obtained from the North Staffordshire and Central Cheshire Research Ethics Committees for all stages of this study.

### Patient Measures

Pain intensity was measured by calculating the mean of three numerical rating scales (0–10, where 0 indicates “no pain” and 10 means “pain as bad as it could be”) for the patient's least, usual, and current (at the time of filling in the questionnaire) levels of LBP over the previous 2 weeks. Higher scores indicate higher levels of reported pain intensity [23–25]. Cronbach's alpha testing of internal consistency (alpha = 0.93) was good for these variables. Incongruence between partners on the ratings of pain intensity have been shown previously [13], therefore we chose to use patient ratings of pain intensity, as such ratings would be less likely directly influenced by the partner's current mood state.

### Partner Measures

The Hospital Anxiety and Depression Scale (HADS) was used to assess the partner's level of depressive symptoms. The HADS contained seven items of depressive symptoms; item scores ranged from 0 to 3; scale scores ranged from 0 to 21, and higher scores indicated greater levels of depressive symptoms [26]. Cronbach's alpha testing showed acceptable levels of internal consistency (alpha = 0.75).

The Revised Dyadic Adjustment Scale (RDAS) [27], a shortened measure of the Dyadic Adjustment Scale (DAS) [28], was used to measure relationship quality and was chosen because of its ease of use, brevity, and convenience to participants. The responses can be scored in three subscales: cohesion (sharing ideas, working on things together, level of discussion and communication, and engagement of outside activities; range 0–19), consensus (agreement on sex relations, affection, religion, and major decisions; range 0–30), and satisfaction (level of disagreements and quarrelling, arguments, and thoughts on separation; range 0–20). A higher score indicates greater levels of these constructs between couples. Subsequent studies have shown the RDAS to be able to discriminate between distressed and nondistressed couples [29] and demonstrate good validity; Cronbach's alpha (0.9), split half testing (0.94). Versions of this measure have been used within chronic back pain populations previously [8,15]. Testing of these variables within this cohort show acceptable to good levels of internal consistency using Cronbach's alpha testing (overall scale = 0.87; cohesion = 0.82; consensus = 0.75; satisfaction = 0.85).

### Patient and Partner Measures

Information on gender, age, and length of relationship (years/months) were collected from both patients and their partners.

### Statistical Analysis

Pearson's correlations were first used to describe the associations between the partner‐rated constructs of relationship quality, patient‐rated pain intensity, partner's depressive symptoms and other variables. The association of patient‐rated pain intensity and partner‐rated relationship constructs were tested using three separate hierarchical linear regression models, one for each of the relationship constructs (cohesion, consensus, satisfaction). Each regression model contains four hierarchical analysis steps. Step one tested the direct unadjusted association of patients' rated pain intensity with the partner‐rated relationship quality construct. Step two adjusted the step one analysis for partner depressive symptoms. Step three adjusted for any interaction between partner depressive symptoms and patient's pain intensity. Finally step four carried out adjustment for partner age, gender, and the length of time (in years) of the relationship between patient and partner. This stepped model was chosen in order to consider direct associations between patient‐rated pain intensity and partner‐rated relationship quality constructs, to adjust for the potential influence of depressive symptoms on judgements of relationship quality (as has been shown in previous literature [8,15]), to consider whether partner depressive symptoms interact with patient's pain intensity, and for the adjustment of potential confounders (e.g., age, gender, length of relationship between partners).

Interpretation of interaction product terms within regression analysis can be difficult when using continuous variables. One suitable way to understand such interaction, post hoc, is to stratify the variables that interact (in this case patient pain intensity, partner depressive symptoms) [30]. Hence patient pain intensity was dichotomized with a score of 5 or more indicating a high pain group. Similarly depressive symptom scores were dichotomized based on a nonclinical population mean score for the HADS depression scale (mean score 3.68) indicating high and low depression groups [31]. Also for the purpose of illustrative clarity a figure (Figure 1) and a table (Table 4) were created using these dichotomized classifications for patients' pain intensity and partners' level of depressive symptoms. Analysis was performed using SPSS version 19 (SPSS, Inc., Chicago, IL).

## Results

### Participants

In total, 299 patients indicated they had a current partner, and 71 (24%) partners responded. We have no information on how many patients passed on partner questionnaires to their partner. Patients with partners who responded were more likely to be female (67% vs 61%), were significantly older (57 years vs 53 years), reported a significant, longer length of relationship with their current partner (32 years vs 25 years), and reported significantly higher levels of pain intensity (mean 3.2 vs 2.4). The mean ages of partners (58.9 years) and patients (58.1 years) were similar. Gender distribution showed that 69% of patients were female, this corresponded to 69% of the partners being male; there was no same‐sex relationships reported within this cohort. Partners indicated that they had been in a relationship with the patient for a mean of 32 years. Paired sample *t*‐tests showed no significant differences between patients and partners on their age (*t* 1.58; *P* = 0.12, two‐tailed), and no significant differences between patients and partners on their judgements of relationship length (*t* 1.20; *P* = 0.23, two‐tailed). Table 1 outlines the characteristics of patients and partners within this study.

**Table 1:** Patient and partner characteristics

	Percentage %	Mean	Standard deviation	Reported minimum/maximum scores
Demographics				
Patient age (years)		58.1	7.2	40–66
Partner age (years)		58.9	8.6	30–81
Patient gender (female)	69%			
Partner gender (male)	69%			
Relationship length (years)		32.4	9.9	7–46
Patient measures				
Pain intensity		3.2	2.8	0–10
Partner measures				
Depression (HADS)		3.6	3.5	0–14
Cohesion		11.3	4.2	2–19
Consensus		24.1	4.0	13–30
Satisfaction		16.0	2.7	7–20

HADS = Hospital Anxiety and Depression Scale.

### Correlation Analysis

Table 2 describes the correlations between the variables within this study. Results show significant positive correlation between the relationship quality variables, and a significant negative correlation between the relationship quality variables and partner‐reported depressive symptoms. Patient‐rated pain intensity had no significant bivariate correlation with any of the partner‐rated variables.

**Table 2:** Pearson's correlations of patient and partner variables

	Pain intensity^‡^	Cohesion^†^	Consensus^†^	Satisfaction^†^	Depressive symptoms^†^	Relationship length^†^	Age^†^
Cohesion^†^	−0.10						
Consensus^†^	−0.12	0.54**					
Satisfaction^†^	−0.12	0.41**	0.62**				
Depressive symptoms^†^	0.20	−0.59**	−0.53**	−0.35**			
Relationship quality length^†^	0.01	0.03	−0.01	0.01	0.00		
Partner age	0.03	0.04	0.06	0.18	−0.12	0.73**	
Patient age	−0.01	−0.00	0.03	0.06	−0.04	0.80**	0.87**

* *P* < 0.05 (two tailed); ** *P* < 0.01 (two tailed).

^†^Partner‐rated.

^‡^Patient‐rated.

### Regression Analysis

Results at step one (i.e., main effect of the association between patient's pain intensity and the partner‐rated constructs of relationship quality) found no direct unadjusted association between patient pain intensity and any of the partner‐rated relationship constructs (cohesion β −0.07, *P* = 0.54, consensus β −0.09, *P* = 0.47, satisfaction β 0.14, *P* = 0.28). Adjustment for partner‐rated depressive symptoms at step two showed no change in the nonsignificant associations between pain intensity and the relationship quality constructs, with partner depressive symptoms being the only consistent variable to be associated with all partners' rated relationship quality variables (i.e., those who report higher levels of depressive symptoms report lower levels for all relationship quality constructs). Results at step three (i.e., interaction effects between patient pain intensity and partner depressive symptoms) show for the cohesion model, no association of patient pain intensity and partner ratings of cohesion, and no association for the interaction between patient pain intensity and partner depressive symptoms. Only partner‐rated depressive symptoms associated with cohesion, with higher levels of depressive symptoms being associated with lower ratings of cohesion. Similar results were found for the satisfaction model. However, results for consensus indicated a significant association between patient pain intensity and partner ratings of consensus, with the direction being positive (i.e., increased patient pain associated with increased partner ratings of consensus). As with the models for cohesion and satisfaction, partner depressive symptoms were negatively associated with consensus. There was also a significant association between the interaction term (combined effects of patient pain intensity and partner depressive symptoms) and consensus, the direction was negative (i.e., the higher the level of patients' pain intensity and partners' depressive symptoms within the interaction, the lower rating of partner consensus). The inclusion of confounders in the final step of the regression models (e.g., age, gender, relationship length) did not significantly alter the reported associations. Model fit testing (ANOVA F tests for regression models) showed that all models were not significant at step 1 (inclusion of pain intensity), but all models were significant at all subsequent steps. Table 3 outlines the results of the final regression analysis for each of the relationship quality constructs.

**Table 3:** Final stage linear regression analysis for spouse relationship variables^a^

Regression model	Predictors	β	95% CI	*P*	Final model R^2^ adjusted, (Final model ANOVA test)
Cohesion	Patient pain intensity	0.002	−0.45 to 0.45	0.99	0.38 (F 7.22, *P* < 0.001)
	Spouse depression	−1.03	−1.53 to −0.53	<0.001	
	Interaction spouse depression and patient pain intensity	0.04	−0.06 to 0.14	0.40	
	Years in relationship	0.08	−0.05 to 0.21	0.21	
Consensus	Patient pain intensity	0.54	0.17 to 0.90	0.005	0.54 (F 13.15, *P* < 0.001)
	Spouse depression	−0.46	−0.88 to −0.04	0.031	
	Interaction spouse depression and patient pain intensity	−0.11	−0.19 to −0.03	0.01	
	Years in relationship	0.01	−0.09 to 0.12	0.81	
Satisfaction	Patient pain intensity	0.08	−0.25 to 0.41	0.62	0.23 (F 4.01, *P* = 0.002)
	Spouse depression	−0.62	−0.98 to −0.25	0.001	
	Interaction spouse depression and patient pain intensity	0.05	−0.02 to 0.12	0.17	
	Years in relationship	−0.06	−0.15 to 0.04	0.24	

a Adjustment for spouse age, spouse gender.

β = Beta Coefficient; 95% CI = 95% confidence interval; F = F distribution test of model fit; *P* = significance level.

**Table 4:** Mean relationship quality scores stratified by partner depression and patient pain grouping

Relationship quality variable	Depression group	Pain group	Mean RQ score	SD	95% CI
Cohesion	Low depression	Low pain	13.0	3.4	11.8–14.2
		High pain	13.8	4.1	9.6–18.1
	High depression	Low pain	9.0	3.6	7.2–10.8
		High pain	8.1	3.6	4.5–11.6
Consensus	Low depression	Low pain	25.4	2.2	24.6–26.1
		High pain	27.7	1.4	26.2–29.1
	High depression	Low pain	23.1	4.4	20.9–25.3
		High pain	17.6	4.7	12.7–22.5
Satisfaction	Low depression	Low pain	16.3	2.1	15.6–17.1
		High pain	18.2	1.5	16.6–19.7
	High depression	Low pain	14.9	3.6	13.1–16.7
		High pain	14.5	2.6	11.8–17.2

CI = Confidence Interval; SD = Standard Deviation; RQ = Relationship quality.

**Figure 2:**
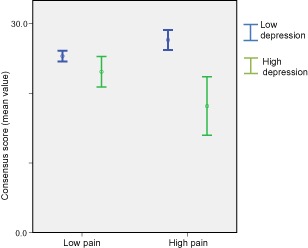
Mean value with 95% confidence intervals of partner consensus stratified by pain and depression.

Post hoc analysis was carried out on the main effect to explain the significant interaction results found within the consensus regression. A graph (Figure 2) was created to illustrate the interaction effect between partner‐rated consensus, partner depressive symptoms, and patient pain intensity using dichotomized scores for patient‐rated pain intensity and partner‐rated depressive symptoms (see methods for criteria of dichotomization). The figure shows that partners with lower levels of depressive symptoms rate their level of consensus higher when the patient they are with reports higher pain, whereas if a partner reports high levels of depressive symptoms, having a partner with high pain leads to a much lower rating of consensus. This shows that pain intensity appears to be associated with greater feelings of consensus, if the partner does not have a high level of depressive symptoms or, as within the regression analysis, where partner depressive symptoms that interact with patients' pain intensity are controlled. However, where partners do report depressive symptoms, the presence of high pain in the patient is associated with much lower feelings of consensus between partners.

To explain the overall effects of patient pain intensity for all relationship quality constructs, a table was created classifying patients into high and low pain groups, and partners classified as low and high depression (Table 4). The table gives mean scores, standard deviations, and 95% confidence intervals. The table shows that partners classified as a high depressive symptom group report lower relationship quality construct scores overall, indicating that depressive symptoms appear to affect ratings of relationship quality. Interestingly partners with low levels of depressive symptoms, who also have a partner with high pain, report higher relationship quality scores for all constructs, compared to partners with low levels of depressive symptoms who have spouses classified as low pain. This indicates that where partners have low levels of depressive symptoms, the level of relationship quality can be elevated in the presence of higher pain within the patient. The lowest overall ratings for relationship quality variables come from partners with high levels of depressive symptoms who also have partners who report high pain.

## Discussion

This study sought to investigate whether pain intensity, as rated by patients with LBP, was associated with their partner's ratings of cohesion, consensus, and satisfaction. Specifically, we wished to test for direct associations between these variables but also account for the influence of partner depressive symptoms, first as an influence on judgements of relationship quality and second in interaction between patient pain intensity and partner depressive symptoms.

This study has shown that there was no direct main effect between patient pain intensity and partner‐rated relationship quality variables of cohesion, consensus, and satisfaction. However, a suppression effect [32] occurred within the consensus model when the interaction term (patient pain intensity and partner depressive symptoms) was introduced. The combined association between patient pain intensity and partner depressive symptoms (i.e., patient pain and the partner depressive symptoms associated with it), when controlled within the regression, revealed a significant positive association between patient pain intensity and partner's rating of consensus. Further post hoc analysis using stratification supports this effect by showing that partners with low levels of depressive symptoms report greater feelings of consensus between themselves and their partner when their partners report higher levels of pain intensity. Similar nonsignificant trends were also found for the constructs of cohesion and satisfaction. Moreover, the findings also show that partners who report high levels of depressive symptoms and are in a relationship with someone with high pain, report the lowest ratings for the relationship quality variables, again particularly notable for consensus.

The findings of this study concur with the general findings that increases in psychological distress, such as depression, are associated with reports of lower levels of relationship quality for partners of those with pain [10,33]. Similarities are also found in comparison to studies reporting patient measures of relationship quality. For example, Waxman et al. [8] found that the association between patient pain intensity and patient‐rated relationship quality was mediated by the level of depression within the patient. Campbell et al. [15] report that after controlling for patient depression, the actual level of patient‐rated relationship quality increased with increasing pain intensity. This current study has found a similar effect using partner ratings for relationship quality. This may indicate that couples could actually feel closer, in terms of consensus (e.g., affection, sexual relations) when one also reports high pain, if the partner does not have elevated depressive symptoms and have depressed symptoms relating to the patient's pain. However, it must also be stated that there may have been many other reasons why partners were depressed that we have not considered, and further work is needed to examine the direct role of patient pain intensity on their partner's depressive symptoms.

While the findings on consensus are of interest, one cannot overlook the issue that the interaction effect was not found for partner ratings of cohesion or satisfaction. This is despite both cohesion and satisfaction having similar correlation values to both pain intensity and depressive symptoms, and therefore the findings could be construed as chance effects. However, we suggest reasons why this might not be the case: first, the general trend for the interaction effect (i.e., construct of relationship quality increases in association with pain intensity once the interaction between depressive symptoms and pain intensity is controlled) can be found for all relationship quality constructs within Table 4. Second, similar effects for consensus (as compared to cohesion and satisfaction) were found in another independent study of primary care patients with LBP using patient‐rated measures [15]. It may be that the construct of consensus (levels of affection, sexual relations) is an important factor in the relationship quality between couples where one has pain.

The theoretical model by Cano and Leong [16] may offer further insight into these findings. A key feature of the model is the interaction process between couples where one has pain. They suggest that patient pain behaviors trigger intrapersonal processes in their partners to help them understand their partner's pain behavior. Partners may interpret the pain behavior negatively, leading to anger and distress underpinned by feeling helpless, feeling sorrow, and having a need for their spouse to be pain‐free. However, there may also be a positive interpretation by the partner, whereby they will have an empathic response with feelings of empathy and a need to offer support to their partner, and this may lead to greater feelings of intimacy and closeness between couples. Our findings suggest that if there is no level of depressive symptoms associated with the patient's pain, and the partner does not have significant levels of depressive symptoms, the actual level of consensus can be elevated by the presence of pain. Of course, offering causality explanations within a cross‐sectional design is speculative, and it may also be that increased feelings of cohesion, consensus, and satisfaction are operant influences on patient pain behavior leading the patient to increase their ratings of pain intensity, similar to the effect of solicitous responses, or that partners with higher consensus perceive their partner to have higher levels of pain. Further longitudinal studies inclusive of information on causative change between these variables would offer greater insight on these potential influences.

Even though this study has shown that relationship length did not play a significant role within the regression analyses, it may still be an important factor. This study included partners with a mean average relationship length of 32 years, the minimum relationship length being 7 years. Added to that is this cohort of patients reported the presence of LBP over 5 years previously, which is suggestive that both themselves (patients) and their partners have been exposed to, and to some extent are still exposed to the influence of pain. Research suggests that couples, where one has a chronic illness, can over time adapt and accommodate to the illness and that this can have a beneficial effect on the relationship [15,34,35]. However, research has also shown that when someone first encounters back pain, the potential initial consequences of their back pain (e.g., losing one's job, changing roles within the family, disability) may have a detrimental effect on the relationship with their partner [4]. It may well be that the effects of pain on partners differ at the outset when someone first experiences pain and further longitudinal research is needed to understand the developmental interaction between pain and relationship quality.

A key strength of this study is the use of data provided by both the patient and their partner as this gives a broader view point of the potential cross‐over between patient‐rated variables and partner‐rated variables. Many previous studies have reported only patient's perspectives on relationship quality which may be influenced by the patient's mood state and level of pain [8,11,15]. However, some previous studies have demonstrated distinct levels of incongruence between partners in their ratings of pain intensity and disability [13,14]. Therefore, the results of this study may well have been different had we used partner‐rated pain intensity or included adjustment for patient mood state. Furthermore, this study would have benefitted from the inclusion of patient‐rated relationship quality variables, as this would have indicated congruence between partners and revealed better the role of the partner's depressive symptoms on such congruence.

A further strength of this study is the inclusion of patients and their partners from a primary care sample. Many previous studies, which have included partners, have included patients from secondary care settings (hospital patients) or via media advertisements which may have limited generalizibility to community samples [5,9,36,37]. In addition, this current study shares similar relationship quality scores compared to other independent cohorts of pain patients and partners [15,38]. Another advantage of this study is the information we have found on the specifics of relationship quality (i.e., constructs of consensus, cohesion, and satisfaction) as this gives an indication of the possible important aspects of relationship quality, between patient and partner (e.g., consensus), that are more likely affected by the presence of pain.

A weakness of the study is the low response rate for partners (24%). We have no way of ascertaining how many partners actually refused to take part as we relied on the patient passing a partner questionnaire over to their partner. It may be that partners with lower relationship quality did not wish to participate. Examining the differences of patient outcomes between those who had a partner who responded and those who did not respond showed that included patients were older, had lived with their partner longer, and reported a greater level of pain intensity. This may have reduced the generalizibility of the results, and further research within different cohorts would be needed to establish if such effects are similar for couples who are younger and/or have less relationship time with their partner. This study is also cross‐sectional, and therefore, we cannot make any assumptions about cause and effect. While stratification is a valid way of investigating interaction analyses [30], the process for this study involved the dichotomization of continuous scale data. The cut‐off points chosen were arbitrary and it is always problematic to interpret data where there can be large variations within each grouping (i.e., data scores around the mean are separated and placed alongside data at the extremes). Therefore, it should be noted that the post hoc analysis such as this was exploratory, and further work would be needed to establish whether effects reported are robust.

Increasing consideration has been given to the inclusion of family members (e.g., partners) in the treatment of those with chronic illnesses [39]. Evidence suggests treatments involving family members can have positive effects on patient outcomes [39–41]. Cano and Leong [16] state the positive effects on patients are indirect. For example the inclusion of a partner in the treatment of someone with pain would not necessarily reduce the patient's pain, but through improved relationship quality couples may cope better, have better empathic understanding, and promote beneficial activity. This current research study has shown that patient pain intensity has a complex association with partner‐rated relationship quality, moderated by the partners' mood state, and that consensus appears to be the relationship construct most influenced. Clinicians may well benefit from asking patients and their partners about the possible impact the patients' pain and disability is having on their relationship, especially aspects of consensus between couples (e.g., affection, sexual relations), and to assess whether pain intensity potentially contributes to marital discord. Certainly there is evidence of beneficial effects of having a satisfying relationship where one person reports pain: recent research has shown that maladaptive appraisals of pain (e.g., catastrophizing) can be attenuated by having a supportive partner [42].

In conclusion, this study has shown that patient pain intensity is associated with the relationship quality rating of their partners, significantly an effect is found for the consensus aspect (affection, sexual relations between couples) of their relationship quality. The findings show this effect is elicited and moderated by the partner's level of depressive symptoms, but when this depression is statistically controlled, the relationship between patients' pain intensity and partners' ratings of consensus is positive. The results of this study demonstrate the associations of patients' pain intensity beyond the patient, and that social context should be considered when evaluating the impact of pain.

## References

[1] Borenstein DG, Wiesel SW, Boden SD. Low Back and Neck Pain: Comprehensive Diagnosis and Management, 3rd edition Philadelphia, PA: Elsevier; 2004

[2] Dagenais S, Caro J, Haldeman S. A systematic review of low back pain cost of illness studies in the United States and internationally. Spine J 2008;8:8–20 18164449 10.1016/j.spinee.2007.10.005

[3] Manchikanti L, Singh V, Datta S, Cohen SP, Hirsch JA. Comprehensive review of epidemiology, scope, and impact of spinal pain. Pain Physician 2009;12:E35–70 19668291

[4] Strunin L, Boden LI. Family consequences of chronic back pain. Soc Sci Med 2004;58:1385–1393 14759683 10.1016/S0277-9536(03)00333-2

[5] Geisser ME, Cano A, Leonard MT. Factors associated with marital satisfaction and mood among spouses of persons with chronic back pain. J Pain 2005;6:518–525 16084466 10.1016/j.jpain.2005.03.004

[6] Linton SJ. A review of psychological risk factors in back and neck pain. Spine 2000;25:1148–1156 10788861 10.1097/00007632-200005010-00017

[7] Gatchel RJ, Peng YB, Peters ML, Fuchs PN, Turk DC. The biopsychosocial approach to chronic pain: Scientific advances and future directions. Psychol Bull 2007;133:581–624 17592957 10.1037/0033-2909.133.4.581

[8] Waxman SE, Tripp DA, Flamenbaum R. The mediating role of depression and negative partner responses in chronic low back pain and relationship satisfaction. J Pain 2008;9:434–442 18313363 10.1016/j.jpain.2007.12.007

[9] Romano JM, Jensen MP, Turner JA, Good AB, Hops H. Chronic pain patient–partner interactions: Further support for a behavioral model of chronic pain. Behav Ther 2000;31:415–440

[10] Leonard MT, Cano A. Pain affects spouses too: Personal experience with pain and catastrophizing as correlates of spouse distress. Pain 2006;126:139–146 16860476 10.1016/j.pain.2006.06.022 PMC1894886

[11] Leonard MT, Cano A, Johansen AB. Chronic pain in a couples context: A review and integration of theoretical models and empirical evidence. J Pain 2006;7:377–390 16750794 10.1016/j.jpain.2006.01.442 PMC1890016

[12] Newton‐John T. Solicitousness and chronic pain: A critical review. Pain Rev 2002;9:7–27

[13] Cano A, Johansen AB, Geisser M. Spousal congruence on disability, pain, and spouse responses to pain. Pain 2004;109:258–265 15157686 10.1016/j.pain.2004.01.036 PMC2679672

[14] Cano A, Miller LR, Loree A. Spouse beliefs about partner chronic pain. J Pain 2009;10:486–492 19345155 10.1016/j.jpain.2008.11.005 PMC2695943

[15] Campbell P, Jordan KP, Dunn KM. The role of relationship quality and perceived partner responses with pain and disability in those with back pain. Pain Med 2012;13:204–214 22222190 10.1111/j.1526-4637.2011.01298.x PMC3491634

[16] Cano A, Leong L. Significant others in the chronicity of pain and disability In: Hasenbring MI , Rusu AC , Turk DC , eds. From Acute to Chronic Back Pain: Risk Factors, Mechanisms, and Clinical Implications. Oxford, UK: Oxford University Press; 2012:339–354

[17] Beach SRH, Katz J, Kim S, Brody GH. Prospective effects of marital satisfaction on depressive symptoms in established marriages: A dyadic model. J Soc Pers Relat 2003;20:355–371

[18] Hollist CS, Miller RB, Falceto OG, Fernandes CLC. Marital satisfaction and depression: A replication of the Marital Discord Model in a Latino sample. Fam Process 2007;46(4):485–498 18092581 10.1111/j.1545-5300.2007.00227.x

[19] Foster NE, Bishop A, Thomas E, et al. Illness perceptions of low back pain patients in primary care: What are they, do they change and are they associated with outcome? Pain 2008;136:177–187 18313853 10.1016/j.pain.2007.12.007

[20] Foster NE, Thomas E, Bishop A, Dunn KM, Main CJ. Distinctiveness of psychological obstacles to recovery in low back pain patients in primary care. Pain 2010;148:398–406 20022697 10.1016/j.pain.2009.11.002 PMC2831173

[21] Jordan K, Clarke AM, Symmons DP, et al. Measuring disease prevalence: A comparison of musculoskeletal disease using four general practice consultation databases. Br J Gen Pract 2007;57:7–14 17244418 PMC2032694

[22] NHS Information Authority . The Clinical Terms Version 3 (The Read Codes). 2000 Birmingham UK, *NHS Information Authority*

[23] Jensen MP, Karoly P. Self‐report scales and procedures for assessing pain in adults In: Turk DC , Melzack R , eds. Handbook of Pain Assessment. New York: Guilford Press; 1992:193–213

[24] Dunn KM, Jordan K, Croft PR. Characterizing the course of low back pain: A latent class analysis. Am J Epidemiol 2006;163:754–761 16495468 10.1093/aje/kwj100

[25] Nicholas MK, Asghari A, Blyth FM. What do the numbers mean? Normative data in chronic pain measures. Pain 2008;134:158–173 17532138 10.1016/j.pain.2007.04.007

[26] Zigmond AS, Snaith RP. The hospital anxiety and depression scale. Acta Psychiatr Scand 1983;67:361–370 6880820 10.1111/j.1600-0447.1983.tb09716.x

[27] Busby DM, Christensen C, Crane DR, Larson JH. A revision of the Dyadic Adjustment Scale for distressed and nondistressed couples: Construct hierarchy and multidimensional scales. J Marital Fam Ther 1995;21:289–308

[28] Spanier GB. Measuring dyadic adjustment: New scales for assessing the quality of marriage and similar dyads. J Marriage Fam 1976;38:15–28

[29] Crane DR, Middleton KC, Bean RA. Establishing criterion scores for the Kansas Marital Satisfaction Scale and the Revised Dyadic Adjustment Scale. Am J Fam Ther 2000;28:53–60

[30] Katz MH. Multivariable Analysis: A Practical Guide for Clinicians, 2nd edition Cambridge: Cambridge University Press; 2006

[31] Crawford JR, Henry JD, Crombie C, Taylor EP. Brief report: Normative data for the HADS from a large non‐clinical sample. Br J Clin Psychol 2001;40:429 11760618 10.1348/014466501163904

[32] MacKinnon DP, Krull JL, Lockwood CM. Equivalence of the mediation, confounding and suppression effect. Prev Sci 2000;1:173–181 11523746 10.1023/a:1026595011371 PMC2819361

[33] Angst F, Verra ML, Lehmann S, Aeschlimann A, Angst J. Refined insights into the pain–depression association in chronic pain patients. Clin J Pain 2008;24:808–816 18936599 10.1097/AJP.0b013e31817bcc5f

[34] Gilad D, Lavee Y, Innes‐Kenig O. The structure of dyadic support among couples with and without disability. J Behav Med 2009;32:453–465 19449204 10.1007/s10865-009-9216-5

[35] Burman B, Margolin G. Analysis of the association between marital relationships and health problems: An interactional perspective. Psychol Bull 1992;112:39–63 1529039 10.1037/0033-2909.112.1.39

[36] Flor H, Breitenstein C, Birbaumer N, Fnrst M. A psychophysiological analysis of spouse solicitousness towards pain behaviors, spouse interaction, and pain perception. Behav Ther 1995;26:255–272

[37] Cano A, Weisberg JN, Gallagher RM. Marital satisfaction and pain severity mediate the association between negative spouse responses to pain and depressive symptoms in a chronic pain patient sample. Pain Med 2000;1:35–43 15101962 10.1046/j.1526-4637.2000.99100.x

[38] Romano JM, Turner JA, Jensen MP, et al. Chronic pain patient–spouse behavioral interactions predict patient disability. Pain 1995;63:353–360 8719536 10.1016/0304-3959(95)00062-3

[39] Martire LM, Lustig AP, Schulz R, Miller GE, Helgeson VS. Is it beneficial to involve a family member? A meta‐analysis of psychosocial interventions for chronic illness. Health Psychol 2004;23:599–611 15546228 10.1037/0278-6133.23.6.599

[40] Keefe FJ, Blumenthal J, Baucom D, et al. Effects of spouse‐assisted coping skills training and exercise training in patients with osteoarthritic knee pain: A randomized controlled study. Pain 2004;110:539–549 15288394 10.1016/j.pain.2004.03.022

[41] Abbasi A, Dehghani M, Keefe FJ, et al. Spouse‐assisted training in pain coping skills and the outcome of multidisciplinary pain management for chronic low back pain treatment: A 1‐year randomized controlled trial. Eur J Pain 2012;1:1033–1043 22337646 10.1002/j.1532-2149.2011.00097.x

[42] Holtzman S, DeLongis A. One day at a time: The impact of daily spouse support on pain, negative affect and catastrophising among individuals with rheumatoid arthritis. Pain 2007;131:1633–1655 10.1016/j.pain.2007.04.005 17517474

